# 2,2,7-Trichloro-3,4-dihydro­naphthalen-1(2*H*)-one

**DOI:** 10.1107/S1600536809032772

**Published:** 2009-08-26

**Authors:** Ben Capuano, Ian T. Crosby, Craig M. Forsyth, James K. Shin

**Affiliations:** a Medicinal Chemistry and Drug Action, Monash Institute of Pharmaceutical Sciences, Monash University (Parkville Campus), 381 Royal Parade, Parkville, Victoria 3052, Australia; b School of Chemistry, Monash University, Clayton, Victoria 3800, Australia

## Abstract

The title compound, C_10_H_7_Cl_3_O, obtained as a major byproduct from a classical Schmidt reaction. The cyclohexyl ring is distorted from a classical chair conformation, as observed for monocyclic analogues, presumably due to conjugation of the planar annulated benzo ring and the ketone group (r.m.s. deviation 0.024 Å). There are no significant intermolecular interactions.

## Related literature

For the Schmidt reaction, see: Schmidt (1923 ▶). Lactams and their derived amidines are common structural moieties in a variety of phamaceutical agents (Fylaktakidou *et al.*, 2008 ▶), and are common in anti­psychotics (Capuano *et al.*, 2002 ▶, 2008 ▶). For the conformation of the cyclo­hexyl ring in monocyclic analogues, see: Lectard *et al.* (1973 ▶); Lichanot *et al.* (1974 ▶).
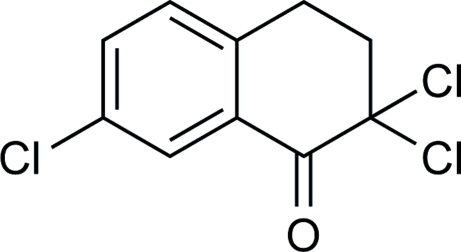


## Experimental

### 

#### Crystal data

C_10_H_7_Cl_3_O
*M* *_r_* = 249.51
Monoclinic, 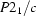

*a* = 8.5233 (1) Å
*b* = 8.0182 (2) Å
*c* = 14.8698 (3) Å
β = 102.561 (1)°
*V* = 991.90 (3) Å^3^
*Z* = 4
Mo *K*α radiation
μ = 0.88 mm^−1^
*T* = 123 K
0.28 × 0.10 × 0.10 mm

#### Data collection

Nonius Kappa CCD diffractometer
Absorption correction: none
9399 measured reflections
2275 independent reflections
1859 reflections with *I* > 2σ(*I*)
*R*_int_ = 0.064

#### Refinement

*R*[*F* ^2^ > 2σ(*F* ^2^)] = 0.037
*wR*(*F* ^2^) = 0.100
*S* = 1.06
2275 reflections
127 parameters
H-atom parameters constrained
Δρ_max_ = 0.53 e Å^−3^
Δρ_min_ = −0.34 e Å^−3^

### 

Data collection: *COLLECT* (Nonius, 1998 ▶); cell refinement: *DENZO–SMN* (Otwinowski & Minor, 1997 ▶); data reduction: *DENZO–SMN*; program(s) used to solve structure: *SHELXS97* (Sheldrick, 2008 ▶); program(s) used to refine structure: *SHELXL97* (Sheldrick, 2008 ▶); molecular graphics: *X-SEED* (Barbour, 2001 ▶); software used to prepare material for publication: *CIFTAB* (Sheldrick, 1997 ▶).

## Supplementary Material

Crystal structure: contains datablocks global, I. DOI: 10.1107/S1600536809032772/hg2549sup1.cif

Structure factors: contains datablocks I. DOI: 10.1107/S1600536809032772/hg2549Isup2.hkl

Additional supplementary materials:  crystallographic information; 3D view; checkCIF report

## Figures and Tables

**Fig. 1.**
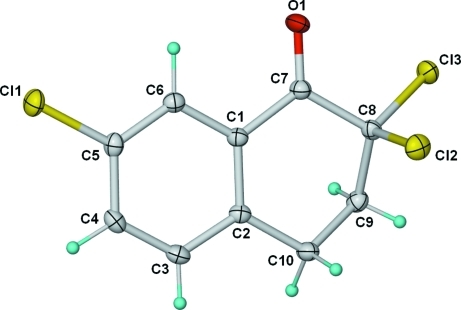
Molecular diagram of the title compound. Displacement ellipsoids are drawn at the 50% probability level.

## supplementary crystallographic information

### Comment

The reaction between hydrazoic acid and carbonyl compounds in the presence of strong acid is known as the Schmidt reaction (Schmidt, 1923) and provides a method for conversion of cyclic ketones to lactams. Lactams as well as their derived amidines are common structural moieties in a variety of phamaceutical agents (Fylaktakidou, *et al.* 2008), but are specifically of interest to our group as they are common in antipsychotics (Capuano, *et al.* 2002, 2008). In the current study, reaction of 7-chloro-1-tetralone with sodium azide and hydrochloric acid gave the desired alkyl migration lactam, 8-chloro-2,3,4,5-tetrahydro-1 *H*-2-benzazepin-1-one, but also a significant amount of the title compound. The solid state structure shows a typical bicyclic ketone framework with two fused six-membered rings and a gem-dichloro substituent in the 2 position. The cyclohexyl ring is distorted from a classical chair conformation, as observed for monocyclic analogues (Lectard, *et al.*, 1973, Lichanot, *et al.*, 1974), presumably due to conjugation of the planar annulated benzo ring and the ketone group (RMS deviation 0.024Å). There are no significant intermolecular interactions.

### Experimental

Sodium azide (1.30 g, 20.0 mmol) was added to a stirred solution of 7-chloro-3,4-dihydronaphthalen-1(2*H*)-one (1.00 g, 5.54 mmol) in concentrated HCl maintained at 0 °C. After warming to room temperature and stirring overnight, the mixture was poured into water and neutralized with K_2_CO_3_. The crude product mixture was extracted with CH_2_Cl_2_ and purified by flash chromatography (silica; ethyl acetate). The fractions containing the title compound were evaporated and the residue was recrystallized from CHCl_3_/hexane yielding beige prismatic crystals. (m.p. 435–436 K). ^1^H NMR (300 MHz, CDCl_3_δ, p.p.m.): 8.12 (d, 1H, *J* = 2.5 Hz, H8), 7.52 (dd, 1H, *J* = 8.0, 2.5 Hz, H6), 7.23 (d, 1H, *J* = 8.0 Hz, H5), 3.18 (t, 2H, *J* = 6.0 Hz, H4), 2.95 (t, 2H, *J* = 6.0 Hz, H3). ^13^C NMR (75 MHz, CDCl_3_δ, p.p.m.): 183.0, 140.4, 134.6, 133.9, 130.3, 129.8, 129.4, 85.7, 43.0, 27.0. *m/z* (EI, 70 ev): 254 (1%, *M*^+^[^37^Cl]_3_), 252 (7, *M*^+^[^35^Cl][^37^Cl]_2_, 250 (24, *M*^+^[^35^Cl]_2_[^37^Cl]), 248 (26, *M*^+^[^35^Cl]_3_), 213 (20), 152 (100), 124 (36), 89 (19). Calcd. for C_10_H_7_Cl_3_O: C 48.1, H 2.8, Cl 42.6; found C 48.1, H 2.9, Cl 42.6%.

### Refinement

All H atoms for the primary molecules were initially located in the difference Fourier map but were placed in geometrically idealized positions and constrained to ride on their parent atoms with C—H distances in the range 0.95–1.00 Å and *U*_iso_(H) = 1.2–1.5 *U*_eq_(C).

### Crystal data

**Table:**

C_10_H_7_Cl_3_O	*F*(000) = 504
*M**_r_* = 249.51	*D*_x_ = 1.671 Mg m^−^^3^
Monoclinic, *P*2_1_/*c*	Mo *K*α radiation, λ = 0.71073 Å
Hall symbol: -P 2ybc	Cell parameters from 9399 reflections
*a* = 8.5233 (1) Å	θ = 2.8–27.5°
*b* = 8.0182 (2) Å	µ = 0.88 mm^−^^1^
*c* = 14.8698 (3) Å	*T* = 123 K
β = 102.561 (1)°	Prism, colourless
*V* = 991.90 (3) Å^3^	0.28 × 0.10 × 0.10 mm
*Z* = 4	

### Data collection

**Table:**

Nonius Kappa CCD diffractometer	1859 reflections with *I* > 2σ(*I*)
Radiation source: fine-focus sealed tube	*R*_int_ = 0.064
graphite	θ_max_ = 27.5°, θ_min_ = 2.8°
φ and ω scans	*h* = −11→11
9399 measured reflections	*k* = −10→10
2275 independent reflections	*l* = −19→19

### Refinement

**Table:**

Refinement on *F*^2^	Primary atom site location: structure-invariant direct methods
Least-squares matrix: full	Secondary atom site location: difference Fourier map
*R*[*F*^2^ > 2σ(*F*^2^)] = 0.037	Hydrogen site location: inferred from neighbouring sites
*wR*(*F*^2^) = 0.100	H-atom parameters constrained
*S* = 1.06	*w* = 1/[σ^2^(*F*_o_^2^) + (0.0478*P*)^2^ + 0.6127*P*] where *P* = (*F*_o_^2^ + 2*F*_c_^2^)/3
2275 reflections	(Δ/σ)_max_ = 0.001
127 parameters	Δρ_max_ = 0.53 e Å^−^^3^
0 restraints	Δρ_min_ = −0.34 e Å^−^^3^

### Special details

**Table:**

Geometry. All e.s.d.'s (except the e.s.d. in the dihedral angle between two l.s. planes) are estimated using the full covariance matrix. The cell e.s.d.'s are taken into account individually in the estimation of e.s.d.'s in distances, angles and torsion angles; correlations between e.s.d.'s in cell parameters are only used when they are defined by crystal symmetry. An approximate (isotropic) treatment of cell e.s.d.'s is used for estimating e.s.d.'s involving l.s. planes.
Refinement. Refinement of *F*^2^ against ALL reflections. The weighted *R*-factor *wR* and goodness of fit *S* are based on *F*^2^, conventional *R*-factors *R* are based on *F*, with *F* set to zero for negative *F*^2^. The threshold expression of *F*^2^ > σ(*F*^2^) is used only for calculating *R*-factors(gt) *etc*. and is not relevant to the choice of reflections for refinement. *R*-factors based on *F*^2^ are statistically about twice as large as those based on *F*, and *R*- factors based on ALL data will be even larger.

### Fractional atomic coordinates and isotropic or equivalent isotropic displacement parameters (Å^2^)

**Table:**

	*x*	*y*	*z*	*U*_iso_*/*U*_eq_	
Cl1	−0.11870 (6)	0.58915 (7)	0.26554 (4)	0.02493 (16)	
Cl2	0.48515 (6)	1.11552 (6)	0.63056 (4)	0.02410 (15)	
Cl3	0.25735 (6)	0.91201 (7)	0.70159 (4)	0.02542 (16)	
O1	0.20373 (18)	1.05238 (18)	0.48477 (11)	0.0256 (4)	
C1	0.2039 (2)	0.7561 (2)	0.48441 (13)	0.0167 (4)	
C2	0.2751 (2)	0.6089 (2)	0.52503 (14)	0.0165 (4)	
C3	0.2196 (2)	0.4562 (3)	0.48443 (14)	0.0199 (4)	
H3	0.2659	0.3555	0.5116	0.024*	
C4	0.0986 (2)	0.4492 (3)	0.40537 (14)	0.0200 (4)	
H4	0.0616	0.3449	0.3786	0.024*	
C5	0.0320 (2)	0.5981 (3)	0.36575 (14)	0.0187 (4)	
C6	0.0818 (2)	0.7514 (2)	0.40393 (13)	0.0180 (4)	
H6	0.0346	0.8514	0.3763	0.022*	
C7	0.2542 (2)	0.9236 (2)	0.52247 (14)	0.0182 (4)	
C8	0.3773 (2)	0.9241 (2)	0.61578 (14)	0.0173 (4)	
C9	0.4939 (2)	0.7787 (3)	0.62603 (14)	0.0200 (4)	
H9A	0.5645	0.7810	0.6884	0.024*	
H9B	0.5627	0.7904	0.5806	0.024*	
C10	0.4060 (2)	0.6127 (2)	0.61131 (14)	0.0198 (4)	
H10A	0.4845	0.5231	0.6079	0.024*	
H10B	0.3583	0.5894	0.6650	0.024*	

### Atomic displacement parameters (Å^2^)

**Table:**

	*U*^11^	*U*^22^	*U*^33^	*U*^12^	*U*^13^	*U*^23^
Cl1	0.0203 (3)	0.0295 (3)	0.0223 (3)	−0.0046 (2)	−0.0012 (2)	−0.0031 (2)
Cl2	0.0264 (3)	0.0186 (3)	0.0248 (3)	−0.0065 (2)	0.0002 (2)	−0.0013 (2)
Cl3	0.0253 (3)	0.0289 (3)	0.0240 (3)	0.0017 (2)	0.0097 (2)	0.0008 (2)
O1	0.0284 (8)	0.0142 (7)	0.0295 (8)	0.0003 (6)	−0.0037 (7)	0.0008 (6)
C1	0.0165 (9)	0.0157 (10)	0.0181 (9)	−0.0002 (7)	0.0043 (8)	0.0014 (8)
C2	0.0156 (9)	0.0158 (10)	0.0188 (9)	0.0010 (7)	0.0054 (7)	0.0017 (8)
C3	0.0206 (10)	0.0152 (10)	0.0250 (10)	0.0018 (8)	0.0070 (8)	0.0012 (8)
C4	0.0207 (10)	0.0168 (9)	0.0240 (10)	−0.0026 (8)	0.0083 (8)	−0.0040 (8)
C5	0.0157 (9)	0.0225 (11)	0.0178 (9)	−0.0038 (8)	0.0035 (8)	−0.0020 (8)
C6	0.0176 (9)	0.0176 (10)	0.0190 (10)	0.0012 (8)	0.0043 (8)	0.0017 (8)
C7	0.0173 (9)	0.0169 (10)	0.0198 (10)	0.0005 (8)	0.0029 (8)	0.0006 (8)
C8	0.0190 (9)	0.0148 (9)	0.0186 (9)	−0.0027 (8)	0.0050 (8)	0.0006 (8)
C9	0.0174 (9)	0.0214 (10)	0.0203 (10)	−0.0007 (8)	0.0021 (8)	0.0012 (8)
C10	0.0208 (10)	0.0157 (10)	0.0215 (10)	0.0012 (8)	0.0018 (8)	0.0027 (8)

### Geometric parameters (Å, °)

**Table:**

Cl1—C5	1.744 (2)	C4—C5	1.396 (3)
Cl2—C8	1.778 (2)	C4—H4	0.9500
Cl3—C8	1.803 (2)	C5—C6	1.382 (3)
O1—C7	1.208 (2)	C6—H6	0.9500
C1—C2	1.402 (3)	C7—C8	1.547 (3)
C1—C6	1.405 (3)	C8—C9	1.518 (3)
C1—C7	1.484 (3)	C9—C10	1.520 (3)
C2—C3	1.401 (3)	C9—H9A	0.9900
C2—C10	1.506 (3)	C9—H9B	0.9900
C3—C4	1.386 (3)	C10—H10A	0.9900
C3—H3	0.9500	C10—H10B	0.9900
			
C2—C1—C6	120.99 (18)	C1—C7—C8	115.33 (16)
C2—C1—C7	122.35 (17)	C9—C8—C7	112.93 (16)
C6—C1—C7	116.65 (17)	C9—C8—Cl2	109.90 (14)
C3—C2—C1	118.42 (18)	C7—C8—Cl2	110.16 (13)
C3—C2—C10	120.16 (17)	C9—C8—Cl3	110.33 (14)
C1—C2—C10	121.41 (17)	C7—C8—Cl3	104.88 (13)
C4—C3—C2	121.35 (19)	Cl2—C8—Cl3	108.46 (11)
C4—C3—H3	119.3	C8—C9—C10	111.50 (16)
C2—C3—H3	119.3	C8—C9—H9A	109.3
C3—C4—C5	118.84 (19)	C10—C9—H9A	109.3
C3—C4—H4	120.6	C8—C9—H9B	109.3
C5—C4—H4	120.6	C10—C9—H9B	109.3
C6—C5—C4	121.79 (19)	H9A—C9—H9B	108.0
C6—C5—Cl1	119.40 (16)	C2—C10—C9	112.99 (16)
C4—C5—Cl1	118.80 (15)	C2—C10—H10A	109.0
C5—C6—C1	118.60 (18)	C9—C10—H10A	109.0
C5—C6—H6	120.7	C2—C10—H10B	109.0
C1—C6—H6	120.7	C9—C10—H10B	109.0
O1—C7—C1	123.49 (19)	H10A—C10—H10B	107.8
O1—C7—C8	121.17 (18)		
